# Levofloxacin Use in Patients with Suspected Tuberculosis in a Community Hospital, Thailand: A Pilot Study

**DOI:** 10.1155/2022/5647071

**Published:** 2022-06-03

**Authors:** Thanawat Khongyot, Sawitree Laopaiboonkun, Throngpon Kawpradid, Kannuwat Jitkamrop, Tawee Chanphakphoom, Suriyon Uitrakul

**Affiliations:** ^1^Department of Pharmaceutical Care, School of Pharmacy, Walailak University, Tha Sala 80160, Nakhon Si Thammarat, Thailand; ^2^School of Tropical Medicine and Global Health, Nagasaki University, Sakamoto, Nagasaki 852-8523, Japan

## Abstract

**Background:**

Levofloxacin is one of the broad-spectrum antibiotics that is indicated for the second-line treatment of tuberculosis (TB). However, using levofloxacin as an empirical therapy for patients without confirmation of TB could still be observed. This descriptive retrospective study, therefore, aimed to investigate the number of levofloxacin use in patients suspected TB in a community hospital in Thailand.

**Methods:**

Patient medical charts of all patients who were admitted to a community hospital in Nakhon Si Thammarat, Thailand, from 2016 to 2017, were reviewed. Patients who were suspected TB and received any levofloxacin-containing regimens were included. Data on patient characteristics and the received regimens were descriptively analyzed and reported as percentage and frequency.

**Results:**

There were a total of 21 patients who received levofloxacin in the hospital. Six of them (28.57%) had the diagnosis of hepatitis. The most prescribed regimen as empirical therapy was levofloxacin, ethambutol, and amikacin (66.67%). After the confirmation of TB using acid-fast bacilli (AFB) test, ten patients (47.62%) still received levofloxacin-containing regimens.

**Conclusion:**

The results from this study indicated high usage of levofloxacin despite no evidence of drug-resistant TB or negative AFB results in a community hospital in Thailand. The results from this study will be further used for the investigation of the prevalence of antibiotic resistance and clinical outcomes of using second-line regimens for TB treatment.

## 1. Introduction

Antimicrobial resistance is one of the most crucial problems worldwide, including in Thailand. The factors relating to antimicrobial resistance include inappropriate use of antibiotics and improper health behavior [1]. Tuberculosis (TB) is a disease caused by bacteria called *Mycobacterium tuberculosis*. These bacteria have been reported approximately 3.4% resistance to antituberculosis drugs in newly diagnosed cases and 19.8% resistance in previously treated cases around the world [2]. Similarly, in Southeast Asian countries, the incidence of antituberculosis drug resistance was 2.1% and 17.2% of new cases and previously treated cases, respectively [2].

According to the World Health Organization (WHO) recommendation, the first recommended regimen for TB is isoniazid, rifampicin, pyrazinamide, and ethambutol (IRZE) [3]. Levofloxacin, based on the recommendation of the Thai National Medicines List, is indicated for moderate to severe community-acquired pneumonia, drug-resistant *Streptococcus pneumoniae* (DRSP), and atypical pneumonia with unable to use macrolides [4]. Thai FDA also suggests that levofloxacin should be preserved for patients with multidrug-resistant tuberculosis (MDR-TB) as a second-line therapy [4]. Moreover, the regimens containing levofloxacin should be preserved for patients with specific indications such as hepatitis or resistance to the first-line TB-medication [3, 4].

Using levofloxacin earlier to the diagnosis of tuberculosis was strongly associated with antituberculosis drug resistance. Also, multiple uses of levofloxacin resulted in lower susceptibility to fluoroquinolones [5]. In addition, patients suspected tuberculosis who received fluoroquinolones significantly had a delay in diagnosis of the disease as compared with patients who did not receive fluoroquinolones [6]. In particular, the immunocompromised patients who received fluoroquinolones prior to the tuberculosis diagnosis had higher risk of antituberculosis drug resistance than those who did not receive [7].

In order to diagnose tuberculosis, there are many methods that can be used. However, the most used method in Thailand is acid-fast bacilli (AFB) staining because of its inexpensiveness and high specificity. On the other hand, the AFB test has been shown to have low sensitivity [8], so some patients might need more than two times of testing to confirm the results. The average number of AFB tests in Thailand ranged between 2 and 3 times per person [9].

The abovementioned results highlighted the important risks of using fluoroquinolones before patients were diagnosed with tuberculosis. In Thailand, there is a lack of studies that indicate the incidence of such usage, as well as its disadvantage. Therefore, this pilot study aimed to investigate the incidence of using levofloxacin in patients who were suspected of tuberculosis without definitive diagnosis of the disease.

## 2. Materials and Methods

This study was a retrospective descriptive study that was performed in a community hospital in Nakhon Si Thammarat, Thailand. Patient medical charts between 2016 and 2017 were reviewed, and the data were recorded. Inclusion criteria in this study were patients who were older or equal to 18 years old, were prescribed levofloxacin in either oral or parenteral dosage form, were suspected *tuberculosis*, and diagnosed using AFB staining. Patients were excluded if they had no information of antibiotic regimens, or were not sent to perform AFB staining after antibiotic prescription.

Patient information was collected including age, gender, main diagnosis, comorbidities (i.e., hepatitis and HIV), AFB staining, bacterial culture (if available), and the prescribed antibiotics. Patients with suspected TB were referred to the patients who were sent to perform AFB staining due to the suspicion of the doctors. Empirical treatment was defined as the antibiotic regimens that patients received on the admission date without any laboratory confirmation of bacterial infection, while definitive treatment was defined as the antibiotic regimens that patients received after bacterial infection was confirmed with laboratory results. In case if patients received the AFB staining test, the antibiotics that they received before the results of AFB were called “pre-AFB regimen,” and the antibiotics that they received after the results of AFB were called “post-AFB regimen.” Hepatitis was defined as patients with alanine transaminase (ALT) and aspartate transaminase (AST) more than three times of the upper normal limit.

All data were analyzed using descriptive statistics including frequency and percentage. The methodology of this study was approved by Human Research Ethics Committee of Walailak University (registration number: WU-EC-PC-2-148-60).

## 3. Results

Between 2016 and 2017, there were 29 patients received levofloxacin in the hospital. Of all, 8 patients did not have AFB staining and were excluded, so a total of 21 patients were recruited for analysis in this study. Baseline characteristics of all patients were described in Table 1. The majority of the patients in this study was men (57.14%) and had age more than 60 years old (57.14%). Six and five patients had been diagnosed with hepatitis and HIV positive, respectively.

Regarding the levofloxacin-based regimens that patients received before the diagnosis of tuberculosis, there were five prescribed regimens of antibiotics in this study (Table 2). Most patients (66.67%) received the regimen of levofloxacin + ethambutol + amikacin. Other drugs that were concomitantly prescribed with levofloxacin and ethambutol in this study included streptomycin, isoniazid, rifampicin, and pyrazinamide. It was found that 2 patients received levofloxacin together with the standard regimen for tuberculosis, i.e., IRZE.

With regard to the patients with diagnosis of hepatitis, none of them received IRZE regimen for empirical therapy. Five out of six patients received levofloxacin, ethambutol, and amikacin, and the other one received levofloxacin, ethambutol, and streptomycin.

Table 3 describes the use of antibiotics after the results of AFB test. Most patients (10 out of 21) still received levofloxacin-based regimens, and seven out of ten patients discontinued using levofloxacin. Two patients unfortunately died prior to the AFB results. For 6 patients with hepatitis, 3 of them still received levofloxacin-based regimens but the other two have changed to the regimens without levofloxacin (Table 4). Unfortunately, one patient died before the AFB results.

## 4. Discussion

The results in this study indicated that many patients in this clinical setting have received levofloxacin-containing regimens as empirical therapy for TB. Only less than 30% of them had hepatitis which might be a condition for levofloxacin use. In addition, although the AFB results were negative, some patients were still administered levofloxacin together with other anti-TB agents.

The use of fluoroquinolones together with ethambutol and amikacin or streptomycin has been observed for decades. However, the recommendation of these regimens was only for patients with specific conditions, e.g., hepatitis and multidrug-resistant TB [3]. Other combinations of levofloxacin could also be observed such as levofloxacin plus IRZE [10], levofloxacin plus RZE [11], and levofloxacin plus amikacin plus IE [12]. Likewise, these regimens were strongly recommended to be preserved for patients with specific conditions and should not be prescribed as empirical therapy prior to the TB diagnosis.

As the spectrum of levofloxacin covers both Gram-positive and Gram-negative infection including drug resistance pathogens, the prescription of this drug should be only for patients with definitive therapy [4]. However, this study reported that approximately 70% of the patients whom the doctors suspected TB received levofloxacin as empirical therapy. Empirical therapy with fluoroquinolones could lead to two serious conditions. Firstly, patients treated with empirical fluoroquinolones tended to have delayed diagnosis of TB [6, 13]. A study reported that patients who received fluoroquinolones had 16-day delay in TB treatment [14]. In the same way, another study showed that patients who had received fluoroquinolones for longer than 5 days had longer duration of TB treatment [15]. Secondly, empirical fluoroquinolones contributed to an increase in the incidence of antituberculosis drug resistance [5].

The results of a study showed that patients who received multiple doses of fluoroquinolones earlier to TB diagnosis tended to have fluoroquinolone-resistant *Mycobacterium tuberculosis* [16]. Furthermore, the characteristics of *M. tuberculosis* should be carefully considered to ensure the penetration of levofloxacin to the target sites [17]. Using levofloxacin earlier to the TB confirmation or using levofloxacin as the first-line regimen in confirmed TB cases could lead to the inappropriate use of antituberculosis drugs and more evidents [11, 18]. Therefore, all health professionals need to emphasize critical decision for empirical therapy in patients who there are some conditions and infections that are suspected of tuberculosis.

Although the use of levofloxacin as an empirical therapy should be considered inappropriate, there are some conditions in that patients may gain a benefit from the drug. Based on the WHO recommendation, patients with isoniazid-resistant TB should receive levofloxacin together with other antituberculosis drugs [19]. Moreover, the patients, who had clinical symptoms indicating TB but had negative AFB results, could get a benefit from levofloxacin continuation because they might have false-negative AFB results due to its low sensitivity [8]. Also, these patients might have other respiratory infectious diseases (e.g., community-acquired pneumonia) that could be treated with respiratory fluoroquinolones such as levofloxacin.

As this study was a retrospective pilot study, several limitations should be considered. Firstly, this study did not yet directly indicate the negative impact of using fluoroquinolones in patients without TB diagnosis. The recent study only highlighted the number of patients who received fluoroquinolones out of the recommendation of the guidelines in a hospital. However, the appropriateness and disadvantage of such usage will be studied in the next phase of our project. Secondly, since this study was a descriptive study, no statistical analysis was performed in this study. Therefore, all numbers should not be inferred to other clinical settings. Lastly, because of the limitation to access to patient data, there were not many parameters that were collected in this study. For example, chest *X*-ray of patients and clinical symptoms of the patient (e.g., fever, weight loss, cough, etc.) were not recorded. Therefore, our future research will try to collect more patient information so that will allow us to statistically analyzed some associations.

## 5. Conclusion

In conclusion, the results in this study indicated the number of patients who received fluoroquinolones before the diagnosis of tuberculosis in a community hospital. Also, some patients were administered fluoroquinolones although they had negative AFB results. These results emphasize the issue of rational antibiotic use, especially in rural areas, and will be used for the future study on the appropriateness of early use of fluoroquinolones and the impact of nonsuitable use of fluoroquinolones.

## Figures and Tables

**Table 1:** Baseline characteristics of patients received levofloxacin-based regimens as empirical treatment for tuberculosis (*N* = 21).

Characteristic	*N* (%)
Male gender	12 (57.14)
Age	
Less than 40 years	5 (23.81)
40–60 years	4 (19.05)
More than 60 years	12 (57.14)
Median age (year)	61.8
Number of acid-fast bacilli (AFB) test	
1 time	3 (14.29)
2 times	6 (28.57)
More than 2 times	12 (57.14)
Diagnosed with hepatitis	6 (28.57)
Diagnosed with HIV infection	5 (23.81)

**Table 2:** The levofloxacin-based regimens that were prescribed as empirical therapy for patients before the diagnosis of tuberculosis. (*N* = 21).

Regimen number	Regimen	*N* (%)
1	Levofloxacin + ethambutol + amikacin	14 (66.67)
2	Levofloxacin + ethambutol + isoniazid + rifampicin	2 (9.52)
3	Levofloxacin + ethambutol + streptomycin	2 (9.52)
4	Levofloxacin + ethambutol + isoniazid + rifampicin + pyrazinamide	2 (9.52)
5	Levofloxacin + ethambutol + amikacin + isoniazid	1 (4.77)

**Table 3:** The use of levofloxacin that were prescribed after the AFB results.

Regimen number	Pre-AFB results (*N*)	Post-AFB results (*N*)		
		Continue levofloxacin	Discontinue levofloxacin	Discontinue all antibiotics
1	14	6	5	1
2	2	1	1	0
3	2	1	1	0
4	2	1	0	1
5	1	0	0	1
Total	21	9	7	3

Two patients in the regimen 1 died before the AFB results were available.

**Table 4:** Clinical data of 6 patients with chronic hepatitis and 2 patients who died before the availability of AFB results.

Patient number	Gender	Number of AFB test	Pre-AFB regimen	AFB results	Post-AFB regimen
1	Female	2	Levofloxacin + ethambutol + amikacin	All negative	Died before AFB results
2	Female	3	Levofloxacin + ethambutol + amikacin	All negative	Continued levofloxacin
3	Male	3	Levofloxacin + ethambutol + amikacin	All negative	Discontinued levofloxacin
4	Male	3	Levofloxacin + ethambutol + amikacin	All negative	Discontinued levofloxacin
5	Male	2	Levofloxacin + ethambutol + streptomycin	All negative	Continued levofloxacin
6	Male	2	Levofloxacin + ethambutol + amikacin	All negative	Continued levofloxacin
7	Male	1	Levofloxacin + ethambutol + amikacin	All negative	Died before AFB results

Patient 1 had chronic hepatitis and died before AFB results.

## Data Availability

The data that support the findings of this study are available from the corresponding author upon reasonable request.
